# Chitosan for the treatment of inflammation of the oral mucosa: A systematic review

**DOI:** 10.4317/medoral.25987

**Published:** 2023-11-22

**Authors:** Cecilia Mónica Zúñiga-López, Kenia Márquez-Pérez, Liliana Argueta-Figueroa, Mario Alberto Bautista-Hernández, Rafael Torres-Rosas

**Affiliations:** 1 Department of Orthodontics, Division of Postgraduate Studies, School of Dentistry, “Benito Juárez” Autonomous University of Oaxaca, Oaxaca, Oaxaca de Juárez, Mexico; 2 National Technological Institute of Mexico/Toluca Technological Institute, Metepec, Mexico state, Mexico; 3 National Council of Humanities, Sciences, and Technologies (CONAHCYT), Mexico city, México; 4 Department of Biosciences, Postgraduate Division, Faculty of Medicine, “Benito Juárez” Autonomous University of Oaxaca, Oaxaca de Juárez, Mexico; 5 Immunology Laboratory, Center for Health and Diseases Sciences Research, School of Dentistry, “Benito Juárez” Autonomous University of Oaxaca, Oaxaca de Juárez, Mexico

## Abstract

**Background:**

Chitosan is a cheap, accessible, nontoxic, biocompatible, and biodegradable compound. Also, this polysaccharide possesses antibacterial and anti-inflammatory properties. Consequently, a wide range of chitosan applications in the dentistry field has been explored. This work aimed to conduct a systematic review to address the clinical efficacy of chitosan for the treatment of oral mucositis.

**Material and Methods:**

The design of the included studies were observational studies, randomized clinical trials (RCT), and non-randomized clinical trials (non-RCT), whereas, a series of cases, *in vivo*, and *in vitro* studies were excluded. The search was performed in PubMed, Web of Science, Scopus, Dentistry and Oral Sciences Source, and ClinicalTrials. Gray literature was searched at Google Scholar. Relevant data from all included studies were recorded. The risk of bias (using RoB 2) and the quality (using Grading of Recommendations Assessment, Development, and Evaluation, GRADE) assessments were carried out.

**Results:**

From the 8413 records screened, 5 clinical trials fully met the eligibility criteria, which comprised a total of 192 participants suffering oral lesions and pain related to oral mucositis. 100% of the included studies exhibited a high risk of bias. The quality of the studies was between low and very low.

**Conclusions:**

The results of the included studies suggest that chitosan can diminish pain and improve the healing of ulcers in oral mucositis. However, there is no conclusive evidence of chitosan as a superior treatment for oral mucositis compared with other current therapies.

** Key words:**Dentistry, chitosan, inflammation, oral disease, wound healing.

## Introduction

Patients with inflammation of the oral mucosa suffer from serious symptomatology, preventing them from eating and drinking, and therefore, affecting their quality of life. Many of these oral pathologies have a severe inflammatory process, such as recurrent aphthous stomatitis, denture stomatitis, oral mucositis, among others, although they have different etiologies (1).

Recurrent aphthous stomatitis (RAS) is the most frequent inflammatory disease in the oral mucosa. This pathology exhibits recurrent, multiple, small, round, or ovoid ulcers that cause considerable pain to the patients. Currently, there is no definitive curative treatment for RAS, and due to that, diverse food supplements, topical treatments, and systemic treatments are used for the treatment of RAS such as antimicrobials, steroids, immunomodulators, and topical barriers (2,3).

Likewise, denture stomatitis (DS) is also an inflammatory disease of the supporting oral tissues that occurs commonly in denture wearers, which is characterized by erythematous lesions and has been associated with the presence of candida species (4). The treatment of DS is based on the use of antifungals. However, there is a rapid recurrence after discontinuation of the treatment and there is an increase in resistance of *Candida* to antifungals. Also, there is a high risk of drug hepatotoxicity (5).

Also, oral mucositis (OM) is an inflammatory disease involving the mucous membranes of the oral cavity. The clinical manifestations of OM are erythematous, erosive, and ulcerative lesions of the oral mucosa, and it is exacerbated by tissue damage produced by sharpened teeth, bruxism, orthodontics appliances, food scraps, and other oral irritants. Thus, subsequent ulcerations become a free entryway for microorganisms (6,7). On another side, OM represents a significant problem for oncological patients, because is one of the most common adverse effects of chemo/radiotherapy for oral cancer (8). Contemporary trends for treating oral mucositis consist of anti-inflammatory drugs, anesthetics, analgesics, antibiotics, cryotherapy, and mucosal coating agents. However, there are no effective options for the treatment of oral mucositis (9,10).

Thus, active compounds from natural sources have attracted the current researcher's interest as an alternative to synthetic medications for the treatment of the most common inflammatory oral pathologies (11).

In that sense, chitosan (poly-N-acetyl glycosaminoglycan) is a natural polysaccharide derived from the deacetylation of chitin. Chitosan has biomedical attributes such as antibacterial, anti-inflammatory wound healing properties. Also, this natural compound is cheap, accessible, nontoxic, biocompatible, and biodegradable (12). In consequence, a wide range of applications in the field of dentistry has been explored.

Concerning the biological functions of chitosan, diverse chitosan-based biomaterials show anti-inflammatory properties such as downregulation of interleukin-1β (IL-1β), interleukin-6 (IL-6), tumor necrosis factor-α (TNF-α) and PGE2. Also, chitosan reduces the phosphorylation of c-Jun N-terminal kinase (JNK), phosphatidylinositol 3-kinase (PI3K), Protein kinase B (AKB), and nuclear factor кB (NF-кB)(13-15). Nevertheless, chitosan diminishes the activities of matrix metalloproteinase 1 (MMP-1), matrix metalloproteinase 2 (MMP-2), caspase 3 (casp-3), and caspase 9 (casp-9), resulting in antiapoptotic properties of chitosan (16).

Several *in vitro* and *in vivo* studies demonstrated that chitosan-based biomaterials can suppress several strains of pathogens, such as bacteria and fungi. For example, chitosan suppresses the resistance properties and hemolytic activity of *Staphylococcus aureus* (17). On the other hand, chitosan inhibits the SAGA (Spt-Ada-Gcn5-acetyltransferase) complex gene expression in *Candida albicans*, which alters the cell surface integrity and their adherence capacity (18). Also, chitosan prevents the fungal mitochondrial biogenesis, leading to a virulence reduction of this strain (19).

Moreover, chitosan-based biomaterials promote wound healing in swab wound incisions of patients undergoing abdominal surgery by providing a suiTable environment for beneficial microbiota such as *Prevotella*, *Lactobacillus*, and Oscillibacter (20). Also, chitosan promotes granulation by inducing fibroblast and keratinocyte proliferation, acting as a progression factor (21). Nevertheless, chitosan modulates the expression of TGF-β and collagen production, improving tissue regeneration (22).

RAS treatment focuses on inhibiting the inflammatory reaction and regenerating the epithelial barrier. On the other hand, DS treatment requires antifungal management. Moreover, OM treatment targets inflammation, tissue damage, and pain control. In consequence, due to the biological functions of chitosan, chitosan-based biomaterials may be a good option for the complementary management of those pathologies.

Dentists should choose the best evidence-based medicine therapy and cost-effective for providing long-term inflammation relief of the oral mucosa. Therefore, we performed a comprehensive systematic review of clinical trials and observational studies to address the efficacy of chitosan in comparison with conventional treatment for oral lesions and pain relief (assessed using the Visual Analog Scale (VAS)) in patients with RAS, DS, and OM.

## Material and Methods

- Study protocol registration

The protocol was registered in the International Prospective Registry of Systematic Reviews (PROSPERO) CRD42022374805. This systematic review was performed according to the Preferred Reporting Items for Systematic Reviews and Meta-Analyses guidelines (PRISMA) and the Cochrane handbook (23).

- Eligibility criteria, Information sources, and Search strategy

Observational studies and clinical trials (randomized or non-randomized) using chitosan as treatment for patients with RAS, DS, and OM were considered eligible for inclusion. Also, the included studies must have a control group (with local treatment or placebo). On the other hand, *in vitro*, animal studies, case reports, observational studies without a control group, reviews, conference abstracts, and articles not indexed in PubMed-Medline were excluded. The included studies must be written in English or Spanish. The eligibility criteria were defined considering the PICO (Population, Intervention, Comparison, and Outcome) strategy. The search was performed on November 2022, without any restriction of publication time, and carried out in the following databases PubMed, Web of Science, Scopus, Dentistry & Oral Sciences Source, ClinicalTrials.gov, whereas gray literature was searched at Google Scholar. Besides, a manual search was performed by reading the references of the included studies. The PICO strategy, review question, and search strategies for each database are shown in Table 1.

- Study selection

The screening of the included studies was performed by reading the title and abstract of each record identified by the search. Subsequently, each full text of the selected articles was acquired and thoroughly reviewed (24).

**Table 1:**
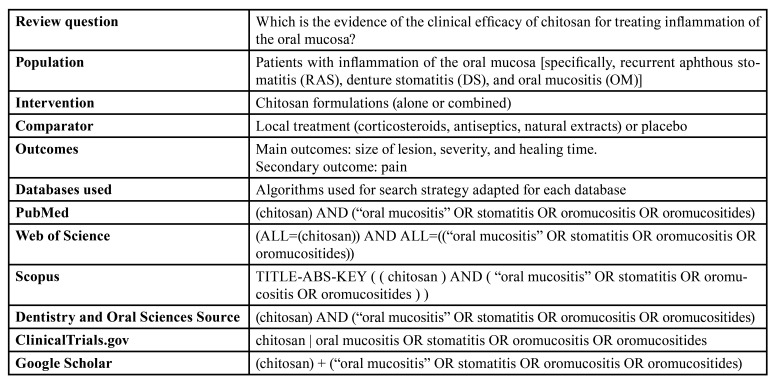
Keywords and algorithms used in the search strategy.

Also, when the reviewed studies did not fully meet the eligibility criteria, these were excluded with reasons.

- Data collection process, Data items, and Data extraction

A standardized Table in Word 2016 (Microsoft Office 355, Microsoft, USA) was prepared for the registration of the relevant data from the included studies, such as study design, population (n), mean and standard deviations of main (size of the lesion/ healing time), and secondary (pain) outcomes. Also, a Table was built to summarize quantitative findings for the main outcome from the included studies. Data extraction was performed independently by two reviewers (CMZL and KMP).

- Synthesis of results

Methodological heterogeneity between the included studies was meticulously analyzed to determine if is possible pooled the data in the quantitative synthesis. However, due to the substantial heterogeneity (clinical and methodological) of the included studies, the quantitative synthesis was not performed. Thus, a qualitative synthesis was carried out.

- Risk of bias and Quality assessments

Two reviewers (CMZL and KMP) performed the risk of bias assessment of the included studies according to the main outcome evaluation (size of lesion/ severity). The disagreements were resolved by consensus of the research group. The Risk of Bias 2 (RoB 2) was used for the risk of bias assessment and the Figure was built as in previous research using the RoB 2.0 Excel tool (25,26). Additionally, the quality of included studies was assessed using the Grading of Recommendations Assessment, Development, and Evaluation (GRADE) criteria.

## Results

**Figure 1:**
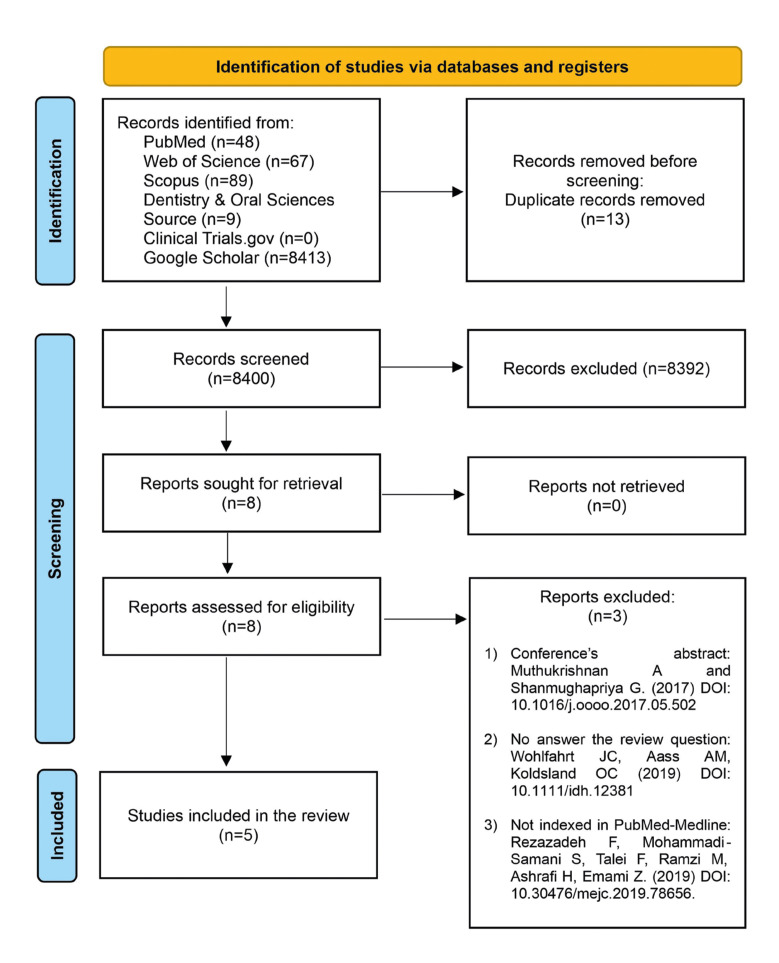
PRISMA flow diagram.

The initial search yielded a total of 8413 records from the databases. Subsequently, after removing duplicates, 8400 records remained. Then, 8 full-text articles were retrieved for eligibility and, of these, 3 studies were excluded with reasons. Thus, 5 clinical trials were included in the present review. The characteristics of the studies, the population, and the study groups are shown in Table 2. The study selection process is detailed in the PRISMA flow diagram (Fig. 1). And the results of the included studies are shown in Table 3.

**Table 2:**
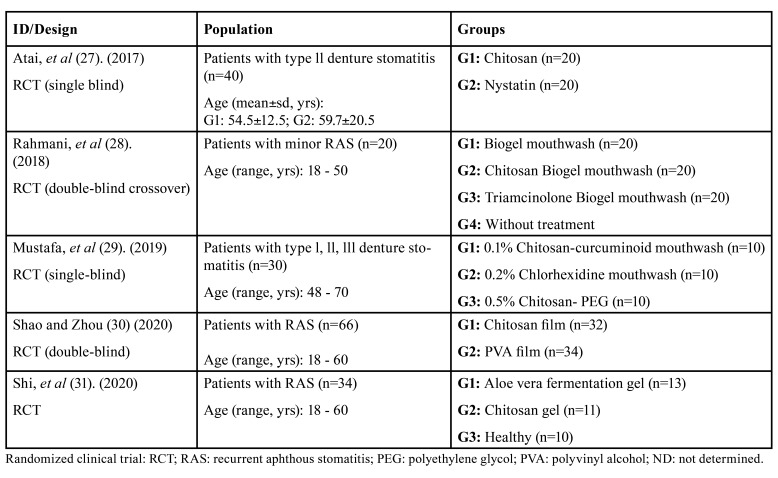
Characteristics of the individual studies.

**Table 3:**
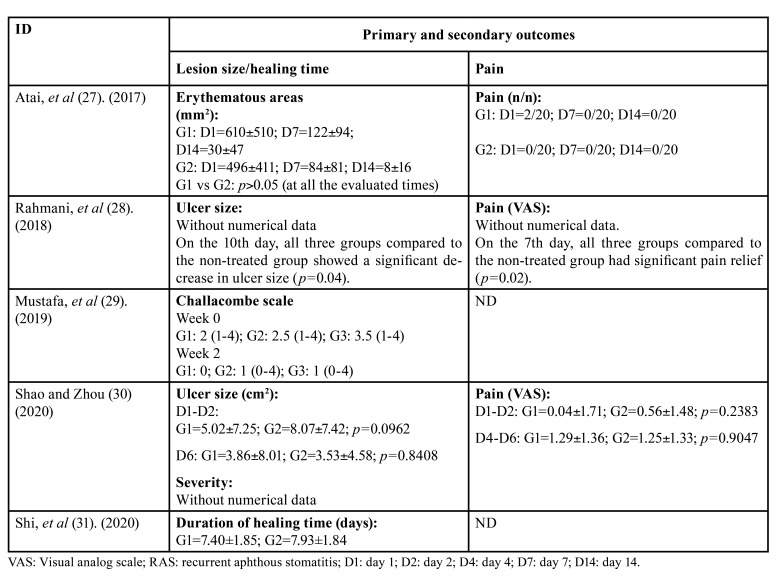
Results of the included studies.

- Chitosan formulations

The chitosan formulations found in the included studies were chitosan solution (concentration at 1%) (27), mouthwash (concentration at 0.5%) (28), mouthwash (concentration at 0.2%) (29), chitosan film (30), and chitosan gel (31).

- Effect of chitosan on the evaluated outcomes

Five studies enrolled 192 participants suffering RAS (28,30,31) or denture stomatitis (27,29). Concerning the oral lesions, 4 articles evaluated the lesion size of OM. Atai, *et al* (27). compared the effect of chitosan vs nystatin on erythematous areas for treating denture stomatitis. The method of application was 2 mL of mouthwash for 2 minutes 4 times a day. The lesion size reduction was statistically similar in both groups on days 7 and 14. In a randomized cross-over study, Rahmani, *et al*. (15) compared the effect of chitosan biogel vs triamcinolone on the ulcer size for treating RAS. The method of application was 5 cc of mouthwash for 4 minutes 3 times a day after each meal. The ulcer size reduction was statistically similar in both groups during ten days of follow-up.

Shao and Zhou compared chitosan vs polyvinyl alcohol film's effect on ulcer size in treating RAS (30). The participants were instructed to use the film twice a day, and the lasting time of the film was about 1 hour. The ulcer size reduction was statistically similar in both groups during six days of follow-up.

Shi, *et al* (31). evaluated healing time in patients with RAS. Chitosan was the intervention group, and the control was Aloe vera fermentation gel, all patients were required to apply a layer of gel on the ulcers 3 times every day until the ulcers disappeared, and found that the healing time was statistically similar in both groups.

Concerning the severity of OM, the Challacombe scale was evaluated in one included study (29). However, the chitosan and the controls showed similar recovery treating OM. Also, regarding pain in patients with OM, three articles (27,28,30) reported that chitosan and their active controls showed pain reduction without statistical differences.

- Risk of bias and Quality assessments

**Figure 2:**
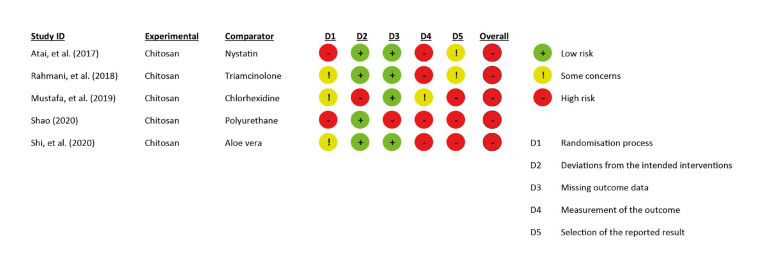
Risk of Bias 2 in the included studies.

All included studies showed a high risk of bias, the main deficiencies were found in the following domains: Randomization process, Measuring outcome, and Selection of the reported results. In the Quality assessment, very low to low certainty of the evidence was observed due to 1) risk of bias and 2) non-assessable consistency between findings in the literature for a single study on the severity of lesion assessment. The risk of bias and quality assessments are shown in Fig. 2 and Table 4, respectively.

**Table 4:**
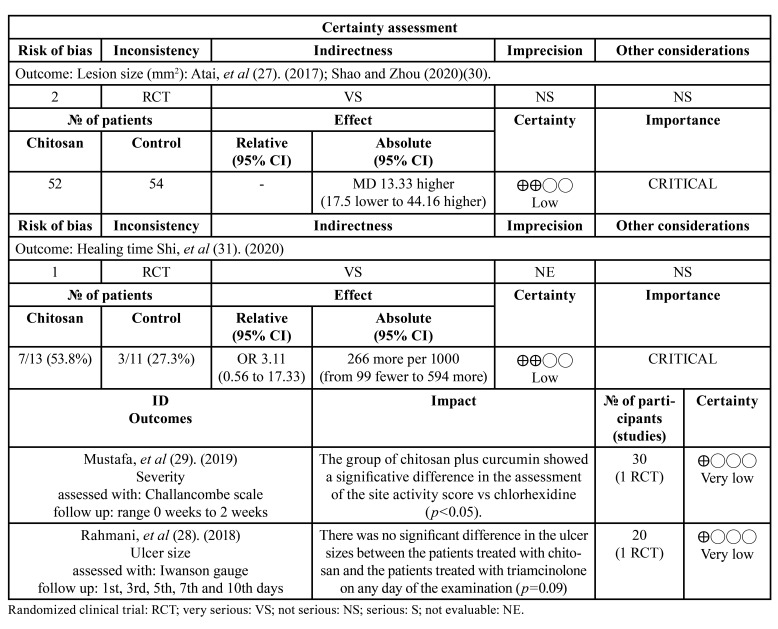
Quality assessment using GRADE.

## Discussion

OM represents a significant problem in oncology due that it is one of the most common adverse effects of drug and radiation regimens or high-dose chemotherapy for cancer treatment with an incidence between 75%-91% (32). Due to the high incidence that occurs in cancer patients, effective treatment is required. The use of benzydamine mouthwash has been reported to be effective in preventing OM. Also, the current treatment of the symptoms in OM patients may include mouthwashes containing topical anesthetics, topical corticosteroids, and benzydamine mouthwash, among others, improving the local pain. On the other hand, coating agents may also provide pain relief by protective coating on the ulcerated oral mucosa. Likewise, these agents have also been used for the treatment of RAS and DM. However, in many cases, the treatment is symptomatic and provides short-term relief. Due to that, in the search for new alternatives, chitosan has been used as a treatment for OM in clinical studies. The included studies showed that the chitosan treatment diminished pain and ulcer size in OM patients. However, the high risk of bias and the low quality of evidence results in a weak recommendation.

Concerning RAS, chitosan properties such as granulation promotion, wound repair, and anti-inflammatory effect seem to diminish ulcer size and pain in patients with RAS in the included studies. Nonetheless, the high risk of bias and the low quality of evidence results in a weak recommendation. Also, the effectivity of chitosan showed inconsistency between the included studies. Similar results were found in the studies in DS.

Chitosan has been investigated by several research groups mainly due to its antimicrobial, biocompatible, anti-inflammatory, and mucoadhesive properties (33). An *in vitro* study reported that chitosan promotes mucosal healing, decreases the production of pro-inflammatory factors, increases the secretion of anti-inflammatory cytokines, and inhibits the intracellular production of reactive oxygen species (34). However, many factors could affect the properties of the chitosan such as factors related to the intrinsic factors related to the material (positive charge density, molecular weight, concentration, hydrophilic or hydrophobic, and chelating capacity, physical state and solubility of the chitosan), conditions of the medium used (ionic state, pH, temperature and time) (35). For instance, biocompatibility seems related with low degree of acetylation (DA) of chitosan (DA<15), high DA is linked with an intense inflammatory response. In this sense, there is some concern about the included studies that did not fully report the characteristics of the chitosan preparation used as intervention, and this may explain the inconsistency of their results.

The limitations of this review include that the chitosan preparations were heterogeneous, as well as other sources of clinical and methodological heterogeneity prevented the results of the included studies from being pooled. On the other hand, the included studies showed high risk of bias. Therefore, it is necessary to carry out more randomized clinical studies to obtain more robust evidence.

## Conclusions

The studies included in this review suggest that chitosan as a treatment for OM, RAS and DS could decrease pain and improve healing of oral lesions. However, the included studies showed a high risk of bias and low quality, which does not allow making a recommendation about their clinical use.
